# Surgical complications in the first and second semesters of the general surgery medical residence. A study of 14063 cases

**DOI:** 10.1016/j.clinsp.2024.100351

**Published:** 2024-04-03

**Authors:** Bruno Henrique Alvarenga, Izabel Cristina Rios, Francisco de Salles Collet e Silva, Edivaldo Massazo Utiyama

**Affiliations:** a Diretoria Clínica do Hospital das Clínicas da Faculdade de Medicina da Universidade de São Paulo (HCFMUSP), São Paulo, SP, Brazil; b Faculdade de Medicina da Universidade de São Paulo (FMUSP), São Paulo, SP, Brazil

**Keywords:** Medical educations, Surgery, Surgical complication

## Abstract

**Objective:**

To evaluate whether the rate of surgical complications is higher during the first semester of the General Surgery residency in the largest hospital complex in Latin America. During this period, students are expected to have less experience in carrying out procedures.

**Methods:**

During a period of two years, all General Surgery resident doctors at the Hospital das Clínicas of the Faculty of Medicine of the University of São Paulo, made a notification of all the procedures they performed (*n* = 14.063), containing information such as name of the procedure, date, who participated, complications, among others. These data were analyzed with the purpose of evaluating the variation in the rate of complications throughout the year.

**Results:**

There was a 52 % increase in the rate of complications in the first academic semester when compared to the second semester. This phenomenon was observed in resident doctors in the first and second years of residency. Furthermore, it was observed that second-year residents remain with high rates of complications, in some procedures, for a longer time than first-year residents. Furthermore, the first three months (March, April and May) seem to have the highest complication rates of the entire year.

**Conclusion:**

The impact of these complications can affect several health services and the increase in surgical complications in the first half of the year must be monitored by institutions, in order to control this phenomenon.

## Highlights

• Surgical complications increase after the entry of new resident doctors.
• Second-year resident doctors need more training time.
• The increase in surgical complications is associated with the entry of new resident doctors, but also with the progression from the first to the second year of the course.

## Introduction

The increase in surgical complications during the period when new resident doctors entered teaching hospitals has been described as the “July effect”. Buchwald et al. 1989, initially associated this term with an investigation into the increase in hospital costs concomitant with the entry of new residents and *fellows.* However, despite the widespread use of the term, their study was unable to prove the increase in costs.^1^ Subsequently, the term became popular and began to be used to explain the increase in hospital complications and was spread with several variations according to the country and the month in which students entered: “July Phenomenon, March Effect, August Killing Season, and Black Wednesday”*.* Some studies showed an increase in mortality at this time when comparing university hospitals with non-university hospitals.^2^^,^^3^ Furthermore, there is also a description of an increase in complications in procedures involving residents.^4^

The reduction in the efficiency of institutions, associated with the change of employees, has been investigated for a long time in the corporate world.^5^^,^^6^ In health services, there is also a particular phenomenon called “cohort turnover”, which consists of the exchange of a large number of employees at a certain time of the year, when less experienced people take over the positions of already trained employees, which can have a negative impact in operations.^7^ In university hospitals, it was observed that this turnover is associated with an increase in the use of resources and in some cases an increase in mortality, which varies with the size and complexity of the organization.^8^ This phenomenon can last for approximately 6 months and can be influenced by the level of local supervision.^8^

However, the literature is not uniform about the existence of this effect, with contradictory results over time in the same research group.^3^^,^^9^ The number of studies that did not demonstrate the existence of the effect is still growing, mainly in research on groups of medical specialties. 10, 11, 12, 13 Therefore, the existence and size of this effect still need to be studied, as well as its implications for medical training and patient care in university hospitals.

In the present study, the objective was to investigate whether there would be a difference between the rates of surgical complications in the first and second semesters in a university hospital located in São Paulo, Brazil. Secondarily, investigate whether this rate would be influenced by the following variables: 1) Difficulty of the procedure (basic or complex), 2) Type of resident participation (main or assistant surgeon) and 3) Year of training (PGY-1 or PGY-2).

## Methods

### Design and subjects

This is a longitudinal, retrospective study, based on monitoring documentation from the general surgery medical residency at the largest hospital complex in Latin America (Hospital das Clínicas da Faculdade de Medicina da Universidade de São Paulo, Brazil). The research period comprised two academic years. The local ethics committee approved the study (CAAE 75160123.0.0000.0068).

During the period studied, the General Surgery residency was carried out during 2 years of in-service training. The definition of semesters did not correspond to the national calendar but to the academic calendar of the residence. The beginning of the first semester was considered to be on March 1^st^ when medical residency traditionally begins in Brazil and the second semester began on September 1^st^ and lasted until February 28^th^ (the date completion of medical residency).

The documentation analyzed consisted of notification forms of each procedure carried out by students in the academic period from March 1, 2018 to February 28, 2020. The first-year student was designated by the acronym PGY-1 and the second-year student by the acronym PGY-2. The period studied does not include the COVID-19 pandemic in Brazil. Notifications of procedures and complications were filled out by resident doctors and the deadline for notification was up to 1 month after the procedure.

### Definition of complications and other variables

Occurrences of surgical complications were recorded by residents on these forms with the following questions: 1) “Did any complications occur with this patient at any time during or after surgery?”, and the open question: 2) “What was the complication?”. To analyze the data, the concept of complication rate was used, which is calculated as the ratio between the number of complications over the total number of procedures performed in the period.

From the records, in addition to the complication rate, the following study variables were created: 1) Difficulty of the procedure (basic or complex), 2) Type of resident participation (main or assistant surgeon) and 3) Year of training (PGY-1 and PGY-2), given the possibility that the rate of complications is influenced by these variables related to the object of the present study.

In the analysis of the variable “difficulty of the procedure”, the data were divided into two categories: 1) Basic and 2) Complex. The basic procedures consisted of minor surgeries such as umbilical herniorrhaphy, orchiectomy and male circumcision, in addition to the procedures generally performed outside the operating room such as central venous access, paracentesis and chest drainage. The complexes consist of procedures such as colon lowering, esophageal coloplasty, hepatectomy and nephrectomy. This division was carried out by medical professors and surgeons experienced in their respective fields.

Complications that could not be confirmed by physical examination were evaluated by complementary examination, such as chest radiography in cases of pneumothorax, Doppler ultrasound for thrombosis, enzymes and electrocardiogram for cases of acute myocardial infarction, cholangiography for biliary fistulas, among others. However, many diagnoses were made clinically by the resident and necessarily confirmed by the supervision of the supervising physician of the surgery department, such as hemorrhage, dehiscence, evisceration, subcutaneous emphysema, among others

All surgical procedures performed with the participation of general surgery residents at the institution were included in the study, including elective or emergency procedures in both the first and second semesters.

### *Statistical analysis*

Statistical analysis was performed using the RStudio cloud and the Jamovi application.^14^^,^^15^ For quantitative variables, the paired *t*-test or Wilcoxon test was performed for variables that did not present a normal distribution. Calculated the sample standard deviation and the 95 % Confidence Interval or the first and third quartile for variables without normal distribution. For qualitative variables, the Chi-Square test *t* was performed.

## Results

Among all procedures, the main complications reported were hemorrhage; infection/sepsis, and dehiscence. Complications such as acute myocardial infarction, stroke, and death were also identified, however, these complications were not the most prevalent in a specific surgical procedure. Among the most frequent complications among all procedures, the occurrence of hemorrhage; infection, and dehiscence were more frequent. The complications of each procedure are described in Table 1.

**Table 1:** Main surgical complications reported by residents.

Main procedure	What was the complication?
Most common in all procedures	Hemorrhage; Infection/ Sepse; Dehiscence
Colon lowering	Bladder injury
Abdominal wall opening	Hollow viscus injury
Central venous access	Difficulty in progressing the guidewire; Pneumothorax; Cephalic progression
Lower limb amputation	Intraoperative arrhythmia
Anastomosis	Fistula; Postoperative ileus; Inferior vena cava injury
Support for polytrauma patients	Hypovolemic shock; Spinal shock; SAH
Pleural biopsy	Subcutaneous emphysema; Lung injury
Access cervicotomy	Venous injury
Cholecystectomy	Gallbladder perforation; Biliary fistula; Vena cava injury; Bradycardia due to vagal reflex during anesthetic induction; Liver laceration; Cystic artery injury, Bile duct injury; Handle drilling
Colectomies	Anastomotic leakage; handle injury
Hypospadias correction	Loss of indwelling urinary catheter
Burn dressing	Total graft loss
Chest drainage	Poorly positioned drain; Lung injury; Intercostal vein injury
Cervical emptying	Internal jugular injury; Nerve injury
Hepatectomies	Inferior vena cava laceration
Giant hernias	Respiratory discomfort after hernia reduction
Incisional hernias	Seroma
Orotracheal intubation	Esophageal intubation
Nephrectomy	Inferior vena cava or renal vein injury
Orchiectomy	Scrotal and penile edema
Pancreatectomies	Prolonged ileus
Laparoscopic pyeloplasty	Urinary fistula
PAI Puncture	Arterial thrombosis
Free flaps	Graft loss
Bladder TURP	Bladder Perforation
Bladder catheterization	Bladder or urethral injury
Abdominal wall suture	Evisceration
Liver transplant	Arterial thrombosis
Tracheostomy	Tracheoesophageal fistula; False route

Note: SAH, Subarachnoid Hemorrhage; TURP, Transurethral Postate Resection; PAI, Invasive Blood Pressure.

The frequency of variables was compared in each of the semesters (Table 2). There was no statistical difference between the semesters regarding the difficulty of the procedure or the type of resident participation. However, a higher proportion of procedures performed by PGY-1 was observed in the second semester. Therefore, it was also decided to carry out an individual analysis of the complication rate among first-year and second-year students, with the aim of removing the influence of this variable in the analysis of the complication rate.

**Table 2:** Characteristics of the procedures carried out in each semester.

	Semester			
	1S (*n* = 6814)	2S (*n* = 7249)	Total (*n* = 14063)	*p-value*
**Difficulty level**				
Basic	5334 (78.3 %)	5613 (77.4 %)	**10947 (77.8** %**)**	0.226
Complex	1480 (21.7 %)	1636 (22.6 %)	**3116 (22.2** %**)**	
**Type of participation**				
Main surgeon	3277 (48.1 %)	3389 (46.8 %)	**6666 (47.4** %**)**	0.111
Assistant	3537 (51.9 %)	3860 (53.2 %)	**7397 (52.6** %**)**	
**Year of training**				
PGY-1	3940 (57.8 %)	4846 (66.9 %)	**8786 (62.5** %**)**	**<0.001**
PGY-2	2874 (42.2 %)	2403 (33.1 %)	**5277 (37.5** %**)**	

Note: 1S, First Semester; 2S, Second Semester; PGY-1, Resident Physician in the first Year of Training; PGY-2, Resident Doctor in the Second Year of Training.

The rate of surgical complications was higher in the first semester of medical residency when compared to the second semester (4.4 % vs. 2.9 %) with statistical relevance demonstrated by the *p*-value < 0.001 (Table 3). The complication rate was 52 % higher in the first semester than in the second.

**Table 3:** Occurrence of surgical complications by semester, difficulty, type of participation and year of training.

		Complication rate (%)	Total patients evaluated	*p*-value
**Semester**	1 - March to August	299 (4.4)	6814	**<0.001**
	2 - September to February	210 (2.9)	7249	
**Difficulty**	Basic	348 (3.2)	10947	**< .001**
	Complex	161 (5.2)	3116	
**Resident's role**	Main surgeon	204 (3.1)	6666	**< .001**
	Assistant	305 (4.1)	7397	
**Year of training**	PGY-1	271 (3.1)	8786	**< .001**
	PGY-2	238 (4.5)	5277	
**Total**		**509 (3.6)**	**14063**	

Note: PGY-1, Resident Physician in the First Year of Training; PGY-2, Resident Doctor in the Second Year of Training.

Basic procedures had a lower complication rate than complex ones (3.25 vs. 5.2 %), consistent with the expected results. The overall rate of complications throughout the study was 3.6 %. This value is closer to the complication rate of basic procedures, this is due to the majority of procedures performed being classified as basic (77.8 %). However, when the resident participated as the main surgeon, it was observed that the number of complications was lower when compared to his role as an assistant (3.1 % vs. 4.1 %).

However, the first and second semesters were not paired by the proportion of procedures with the participation of PGY-1 and PGY-2, and in the second semester, the proportion of procedures performed by PGY-1 was greater than those performed by PGY-2. To define whether this variable could influence the present finding, a separate analysis was carried out for the PGY-1 and PGY-2 groups, with the purpose of comparing whether the complication rate would be higher during the first semester for each of these two groups (Table 4).

**Table 4:** Surgical complication rate per year of resident training.

		Semester					
Year of training	Occurrence of complication	1S (%)		2S (%)		Total	*p-value*
**PGY-1**	No	3789	(96.2)	4726	(97.5)	**8515**	**<0.001**
	Yes	151	(3.8)	120	(2.5)	**271**	
	Total	3940	(100)	4846	(100)	**8786**	
**PGY-2**	No	2726	(94.9)	2313	(96.3)	**5039**	**0.014**
	Yes	148	(5.1)	90	(3.7)	**238**	
	Total	2874	(100)	2403	(100)	**5277**	

Note: 1S, First Semester; S2, Second Semester; PGY-1, Resident Physician in the First Year of Training; PGY-2, Resident Doctor in the Second Year of Training.

Analysis of only PGY-1 showed that the incidence of complications remained higher in the first semester (3.8 % vs. 2.5 %). In the PGY-2 group, it was also observed that the complication rate was higher in the first semester (5.1 % vs.3.7 %).

To better understand how the reduction in complications occurred throughout the training year, a quarterly analysis of the complication rate was carried out, however, as it was not possible to maintain the distribution of paired variables in all quarters, the analyses were stratified by each variable so that they would not influence the quarterly analysis (Table 5).

**Table 5:** Quarterly surgical complication rate by year of training and difficulty of the procedure.

Year of formation	Difficulty	1Q	2Q	3Q	4Q	Total	*p*-value
**PGY1**	**Basic**	4.2 %	2.9 %	2.1 %	2.0 %	**2.7** %	**<0.001**
	**Complex**	4.7 %	5.6 %	3.8 %	4.3 %	**4.4** %	0.579
	**Total**	4.3 %	3.4 %	2.5 %	2.4 %	**3.1** %	**0.001**
**PGY2**	**Basic**	4.5 %	3.4 %	3.9 %	4.2 %	**4.0** %	0.626
	**Complex**	9.4 %	8.5 %	2.8 %	2.3 %	**6.3** %	**<0.001**
	**Total**	5.9 %	4.4 %	3.7 %	3.8 %	**4.5** %	**0.02**
**Total**	**Basic**	4.3 %	3.1 %	2.8 %	2.6 %	**3.2** %	**0.002**
	**Complex**	7.1 %	6.8 %	3.5 %	3.7 %	**5.2** %	**<0.001**
	**Total**	5.0 %	3.8 %	2.9 %	2.8 %	**3.6** %	**<0.001**

Note: 1Q, First Quarter; 2Q, Second Quarter; 3Q, Third Quarter; 4Q, Fourth Quarter; PGY-1, First-year medical residency student; PGY-2, Second-year medical residency student.

In the quarterly analysis, the complication rate continued to decline progressively throughout the annual course of medical residency for PGY-1 and PGY-2 separately. However, when stratifying basic and complex procedures, no statistical difference was observed between complex procedures with the participation of PGY-1 or basic procedures performed by PGY-2, suggesting that the complication rate of these does not change throughout the year of training.

In the general analysis by quarter, a progressive reduction in surgical complications continued to be observed throughout the year. However, there was no observed statistical difference between the total rate of complications between the 2^nd^, 3^rd^ and 4^th^ trimesters. Therefore, the first trimester is the only one that differed from all the others and presented the highest rate of complications (5 %), probably due to the arrival of new residents and the promotion of PGY-1 to PGY-2.

**Fig. 1:**
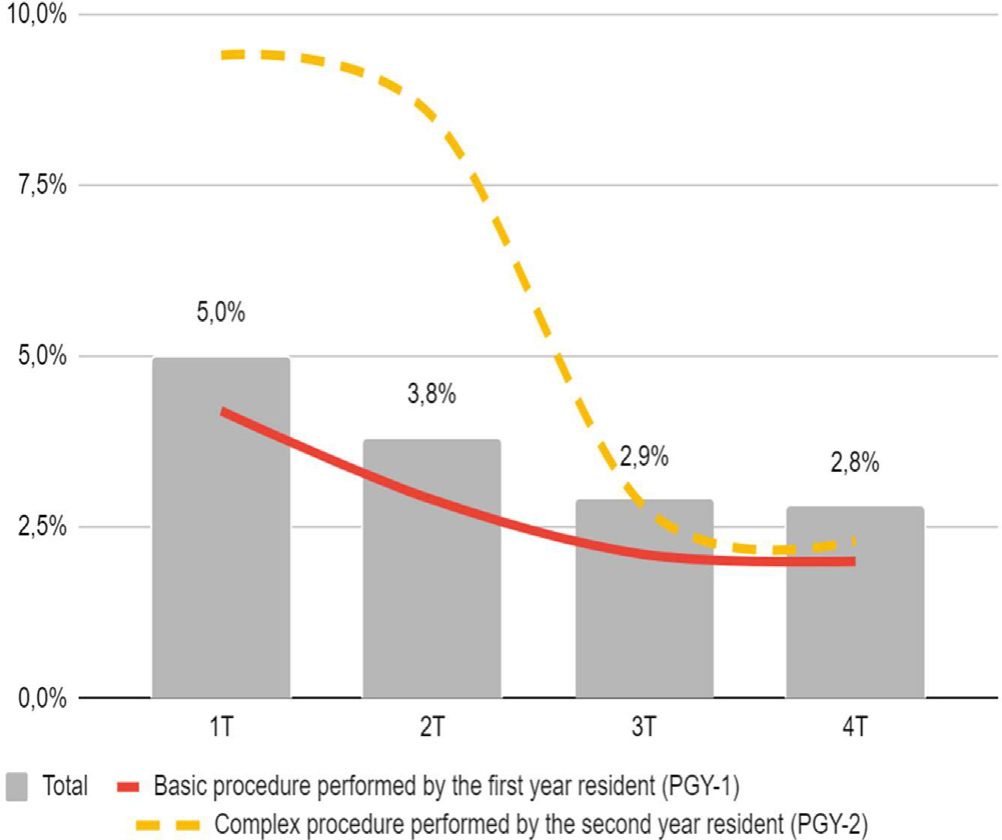
Quarterly surgical complication rate. Note: There is a progressive drop in the rate of complications represented by the gray bars. However, the first quarter is the only one that differs statistically. The individual analysis shows that PGY-2s need two trimesters to show a reduction in the complication rate of complex procedures (dashed yellow line).

Quarterly data suggests that after the first quarter (March to May), the complication rate reduces to its lowest levels. However, it is clear that the complex procedures performed by R2 had the highest complication rates, which only reduced after the 2^nd^ trimester. This finding suggests that special attention should be given to this group in order to mitigate this effect. It is likely that there will be a need for more training time to acquire the PGY-2 knowledge and skills to perform complex procedures (Fig. 1).

## Discussion

In this study, the surgical complication rate was higher during the first semester of medical residency, when compared to the second semester. In the quarterly analysis, the complication rate was higher only in the first quarter and with no statistical difference between the second, third and fourth semesters. However, even in the quarterly analysis, complex procedures with the participation of PGY-2 continued to have high rates of surgical complications in the first two quarters. Most of the procedures performed by the PGY-1 as primary surgeon were basic and his role in complex procedures was generally as an assistant.

The findings presented here are similar to Dasenbrock et al. 2008, which demonstrated an increase in complications in surgery for spinal metastases. Additionally, it also demonstrated an increase in mortality during the period,^18^ which was not evaluated in the present study. It also agrees with the results of the review by Young JQ et al. 2011, which observed a decrease in the efficiency of hospitals during the exchange of residents.^17^ However, it differs from the review by Zogg et al. 2021 which did not demonstrate the presence of the “July effect” among publications to date.^19^

Unlike other studies, this study did not find a high incidence of acute myocardial infarction, although this complication has already been demonstrated as an important post-surgical complication.^21^, ^22^, ^23^ However, some characteristics in patient selection may explain this phenomenon. The present study evaluated patients and procedures of high and low complexity, with low complexity procedures being more frequent, a phenomenon that may explain the fact that cardiac complications were not the most frequent in this study. Furthermore, this study analyzes all surgical procedures performed with the participation of a resident doctor in the specialty of general surgery, including orotracheal intubation, chest drainage, skin biopsy, and debridement of skin wounds, among others whose complication rates are low and/or heart attack of the myocardium is not an expected complication, unlike what is expected from major surgical procedures. Furthermore, the authors did not analyze all procedures performed in the hospital, but only those that were performed with the participation of the general surgery resident, so the complication rates are not intended to represent all surgeries performed, but only those with the participation of resident doctors.

In the stratified quarterly analysis, it was observed that some procedures did not show a reduction in the rate of complications throughout the study period. The first are complex procedures with PGY-1 participation. This occurrence is possibly due to the fact that during this period students did not acquire skills to reduce these complications. The second is the basic procedures performed by PGY-2. This finding was surprising, as it is expected that second-year residents have already acquired skills to perform these procedures during the first year of training. A possible explanation for this phenomenon would be that the basic procedures designated for PGY-2 were generally carried out in more severe patients, or in cases that PGY-1 was not able to resolve. Therefore, it is expected that they will have a higher rate of complications.

Therefore, the higher rate of complications in PGY-2 compared to PGY-1 can be credited to the fact that PGY-2 was assigned procedures with a greater possibility of complications, while PGY-1 was assigned tasks with lower complexity.

The increase in complications may reflect an increase in morbidity and hospital costs, as assessed in the study that disseminated the “July effect”.^1^ The discovery of this effect must be associated with the search for measures to contain this phenomenon. In this sense, the observation that the complication rate in the first semester of residency can potentially affect thousands of patients requires special attention to the medical education of General Surgery residency students.

Some strategies adopted in the literature seem to optimize resident training, such as carrying out simulations to perform procedures.^16^ Another strategy could be greater monitoring of residents in the first months of residency and also when they are promoted to higher levels.^17^

On the other hand, while the explanation of the “July effect” focuses on the entry of new residents in the first year of training,^20^ in the present study, high rates of surgical complications were observed among students in the second year of training (PGY-2), suggesting the presence of other factors explaining the phenomenon. The acquisition of skills among PGY-2s would be slower when compared to the beginning of the training of PGY-1s as it involves cases of greater complexity and severity.

However, it is important to highlight that the study was carried out in a highly complex hospital that drains more complex cases from other hospitals and the referral network for urgent and emergency cases. Therefore, more serious cases are expected with a higher morbidity rate. In addition, it is expected that due to the greater complexity of the cases, the acquisition of surgical skills will take longer than when compared to the same procedure performed in a low-complexity hospital. Another characteristic of this study is that all surgical procedures were analyzed together and possibly the observed effect is not distributed homogeneously among all the procedures studied.

Another limitation of the study is that the notification was carried out by the resident doctors who participated in the procedure, which can cause a bias in the report when compared to an external observer. However, as in the different periods analyzed, the notification was always carried out by the resident doctor, it is believed that this phenomenon has a low impact on the main objective of the study, which is the comparison of complication rates in different periods within the institution. However, the authors recognize that this phenomenon limits the isolated comparison of the complication rates with other studies whose notifications were carried out by external observers.

Regarding the medical literature on this subject, the possibility of publication bias must still be considered, as it is not attractive to publish a publication demonstrating the increase in complications resulting from the entry of new students, a fact that could drive new patients away from the service studied. Therefore, the increase in complications associated with the exchange of residents may be underestimated in the literature, making it difficult to address them in medical education.

## Conclusion

In this study, the rate of surgical complications was higher in the first semester of medical residency. Surgical complications also varied according to the complexity of the procedure, the type of resident participation, and the training period in which they were. The rate of complications was higher in the first semester, even when evaluating each of these variables individually. These findings can be attributed to the greater complexity and severity of the cases treated at the hospital under study and supported improvements in surgery residency programs.

## CRediT authorship contribution statement

**Bruno Henrique Alvarenga:** Conceptualization, Methodology, Formal analysis, Writing – original draft. **Izabel Cristina Rios:** Conceptualization, Investigation, Writing – review & editing. **Francisco de Salles Collet e Silva:** Conceptualization, Investigation, Writing – review & editing. **Edivaldo Massazo Utiyama:** Validation, Writing – review & editing, Supervision.

## Declaration of competing interest

The authors declare no conflicts of interest.
